# Loss of DAP12 and FcRγ Drives Exaggerated IL-12 Production and CD8^+^ T Cell Response by CCR2^+^ Mo-DCs

**DOI:** 10.1371/journal.pone.0076145

**Published:** 2013-10-14

**Authors:** Grzegorz B. Gmyrek, Holly M. Akilesh, Daniel B. Graham, Anja Fuchs, Lihua Yang, Mark J. Miller, Gabriel J. Sandoval, Kathleen C. F. Sheehan, Robert D. Schreiber, Michael S. Diamond, Wojciech Swat

**Affiliations:** 1 Department of Pathology and Immunology, Washington University School of Medicine, St. Louis, Missouri, United States of America; 2 Department of Medicine, Washington University School of Medicine, St. Louis, Missouri, United States of America; 3 Department of Molecular Microbiology, Washington University School of Medicine, St. Louis, Missouri, United States of America; Oklahoma Medical Research Foundation, United States of America

## Abstract

Dap12 and FcRγ, the two transmembrane ITAM-containing signaling adaptors expressed in dendritic cells (DC), are implicated in the regulation of DC function. Several activating and adhesion receptors including integrins require these chains for their function in triggering downstream signaling and effector pathways, however the exact role(s) for Dap12 and FcRγ remains elusive as their loss can lead to both attenuating and enhancing effects. Here, we report that mice congenitally lacking both Dap12 and FcRγ chains (DF) show a massively enhanced effector CD8^+^ T cell response to protein antigen immunization or West Nile Virus (WNV) infection. Thus, immunization of DF mice with MHCI-restricted OVA peptide leads to accumulation of IL-12-producing monocyte-derived dendritic cells (Mo-DC) in draining lymph nodes, followed by vastly enhanced generation of antigen-specific IFNγ-producing CD8^+^ T cells. Moreover, DF mice show increased viral clearance in the WNV infection model. Depletion of CCR2+ monocytes/macrophages *in vivo* by administration anti-CCR2 antibodies or clodronate liposomes completely prevents the exaggerated CD8+ T cell response in DF mice. Mechanistically, we show that the loss of Dap12 and FcRγ-mediated signals in Mo-DC leads to a disruption of GM-CSF receptor-induced STAT5 activation resulting in upregulation of expression of IRF8, a transcription factor. Consequently, Dap12- and FcRγ-deficiency exacerbates GM-CSF-driven monocyte differentiation and production of inflammatory Mo-DC. Our data suggest a novel cross-talk between DC-ITAM and GM-CSF signaling pathways, which controls Mo-DC differentiation, IL-12 production, and CD8^+^ T cell responses.

## Introduction

Signaling through immunoreceptor tyrosine-based activation motifs (ITAM) is an important mechanism to control the activation of dendritic cells (DCs). DCs express two ITAM containing adaptors: DNAX activation protein-12 (Dap12) and FcRγ that channel signals from several immunoreceptors and non-immunoreceptors (including integrins) and use a canonical ITAM signaling module involving Syk kinase, the Vav GEFs, and SLP76 for downstream signal transduction [1]–[3]. The role of dendritic cell ITAM-containing adaptors (DC-ITAM) in modulating immune responses is unclear, since they have been reported to enhance or inhibit immune responses depending on the study. For example, a disruption of DC-ITAM led to enhanced proinflammatory cytokine production after TLR stimulation and an augmented type I interferon response [4]–[5]. Conversely, DC ITAM positively regulates septic shock, reactive oxygen species (ROS) production, phagocytosis, and MHC class II recycling [6]–[8]. Thus, DC-ITAM modulation of TLR, GM-CSF or IFNAR signaling pathways might selectively alter external signals regulating inflammatory effector responses [9]. For example, Dap12 deficiency in mice results in altered activity of antigen-specific T cells [10]–[12]. Moreover, Dap12 and FcRγ deficiency results in complete protection against induction of experimental autoimmune encephalomyelitis (EAE) [8]. Here, we show that Dap12 and FcRγ deficiency enhances endogenous CD8 T cell response to protein antigen or WNV infection. Specifically, a deficiency in ITAM signaling alters GM-CSF-driven induction of IRF8, leading to increased Mo-DC differentiation, followed by upregulation of IL-12 production. Our data provide evidence for cross-talk between ITAM and TLR or GM-CSF signaling pathways, which modulates Mo-DC differentiation and IL-12 cytokine-driven regulation of CD8 T cell responses.

## Materials and Methods

### Mice

Mice deficient in Dap12 and FcRγ (referred to DF mice) have been previously described [7]-[8] and were a gift from Dr. M. Colonna (Washington University, St. Louis, MO). For breeding strategy we used offspring of Dap12^+/−^FcRγ^−/−^ × Dap12^+/−^ × FcRγ^−/−^ mice as previously described [13]. OT-1 and C57BL/6 mice were a gift of Dr. A. Shaw (Washington University, St. Louis, MO). Vav^NULL^ mice (mice deficient in Vav1, Vav2, and Vav3 proteins) have been described [7]–[8]. All mice were kept in Specific Pathogen Free (SPF) conditions and animal experiments were approved and performed according to the Animal Studies Committee of Washington University School of Medicine.

### Reagents

Anti-mouse antibodies (Abs) FITC, PE, APC, PE-Cy5, APC-Cy7, PerCP-Cy5.5, PECy7 - B220, TCRβ, CD4, CD8, NK 1.1, Ter119, CD11b, CD11c, PDCA-1, Ly6C, Ly6G, Vα2, and I-A^b^, were purchased from Becton Dickinson Biosciences, Biolegend, and eBioscience. IL-2 and IFNγ ELISPOT Pair Sets, streptavidin-alkaline phosphatase (AKP) were obtained from Becton Dickinson Biosciences. The phosphoSTAT5 (pSTAT5) antibody was from Cell Signaling. Fetal Calf Serum (FCS) was from Atlanta Biologicals. 2-mercaptoethanol and combination of nitro-blue tetrazolium chloride (NBT) and 5-bromo-4-chloro-3'-indolyphosphate p-toluidine salt (BCIP) known as SigmaFast BCIP/NBT were purchased from Sigma-Aldrich. Dulbecco Modified Eagle Medium (DMEM), sodium pyruvate, penicillin and streptomycin, 100x concentrated nonessential amino acid solution, Fix and Perm reagent set were from Invitrogen. BSA and clodronate liposomes were obtained from Fisher Scientific and Encapsula Nanosciences, respectively. Chicken-derived ovalbumin peptide specific for MHC class I OVA_257–264_ (SIINFEKL) was a gift from Dr. P. Allen (Washington University, St. Louis, MO). MC21 Ab was a gift from Dr. M. Mack (University of Regensburg, Germany). Complete Freund Adjuvant (CFA) was obtained from Difco. GM-CSF and Flt3L were gifts from Dr. M. Colonna (Washington University School of Medicine, St. Louis, MO).

### Mice footpad immunizations

Mice footpad immunizations have been previously described [8], [14]. Briefly, SIINFEKL diluted in PBS and CFA was emulsified with the interchangeable syringes connected with micro-emulsified (18G) needle (Popper & Sons). The prepared emulsion (100 µM solution) was injected subcutaneously in two hind feet at amount of 50 µl to each hind foot. At day 7, popliteal lymph nodes were harvested followed by cell isolation, resuspensed in DMEM-10 media (supplemented with 10% fetal calf serum, 2 mM L-glutamine, 1 mM sodium pyruvate, 100 U/mL penicillin and streptomycin, 1 mL/100 mL media of a 100× concentrated nonessential amino acid solution, and 50 µM 2-mercaptoethanol), counted and processed for further analysis.

### West Nile Virus infection

WT and DF mice were infected subcutaneously in the footpad with 100 plaque-forming units (PFU) of WNV strain 3000.0259 as described previously [15] and subsequently euthanized seven days later. The antigen-specific CD8 T cell response was measured by IFNγ ELISPOT after a recall response to a D^b^-restricted WNV-specific NS4B peptide. Brains from WT and DF mice were harvested after extensive tissue perfusion with phosphate-buffered saline (PBS) at 4°C, and homogenized using a bead beater apparatus, followed by evaluation of virus titers using plaque assay on BHK21-15 cells as described previously [15].

### Cytokine evaluation

For intracellular IL-12 staining, BMDCs were stimulated with 1 µM of CpG1826 for 6 hrs followed by treatment with brefeldin A, and intracellular staining with anti-IL-12p40 using Fix&Perm kit accordingly to manufacturer instructions. For evaluation of IL-12p40 expression *ex vivo*, pre-sorted (to exclude CD3ε, panNK, CD19 and Ter119) CD11c^+^ DCs from draining lymph nodes of immunized mice were stimulated with CpG1826 (1 µM) for 6 hrs in the presence of brefeldin A and fixed and permeabilized cells were stained for IL-12p40 within Mo-DCs (CD11c^+^CD11b^+^Ly6C^+^MHCII^+^) and CD8α DCs (CD11c^+^CD8α^+^). To evaluate IFNγ production by re-stimulated cells isolated from draining lymph nodes, an ELISPOT was employed as described previously [8], [14]. Briefly, 96-plates with immobilon-P membrane were coated overnight with primary IFNγ Ab. Cells isolated from draining lymph nodes (dLNs) were plated at concentration 5×10^5^ cells per well followed by overnight re-stimulation with antigen (SIINFEKL; 10 µM). After removing the cells, plate was developed as described previously [8], [14]. Finally for quantitative evaluation of IFNγ concentration in supernatants after overnight re-stimulation of draining lymph nodes with SIINFEKL (10 µM), the bead-based ELISA (cytometric beads array) described elsewhere [16] was used according to manufacturer’s instructions. To determine the IL-12p40 concentration in supernatants essentially the same immunoassay was employed with BMDC that were stimulated for 6hrs with LPS (10 ng/ml) or CpG1826 (250 nM).

### Bone-marrow dendritic cells derived generation

Bone marrow cells were isolated as previously described [8] and cultured in DMEM-10 supplemented with either 2% granulocyte macrophage colony-stimulating factor (GM-CSF) or Flt3L. At day 5 to 10 the bone marrow-derived dendritic cells (BMDC) were used for indicated cellular assays.

### Flow cytometry

The following combination of antibodies followed by exclusion of the dump gate was used to determine DCs subsets. For conventional dendritic cells (CD8α DCs): CD11c^+^CD8α^+^ (dump gate: CD4, TCRβ, pan NK, Ter119, CD11b, B220), and monocyte-derived dendritic cells (Mo-DCs): CD11c^+^CD11b^+^Ly6C^+^Ly6G^−^ MHCII^+^ (dump gate: CD4, CD8α, TCRβ, pan NK, Ter119, B220). For DC isolation, the lymph nodes were treated with collagenase D followed by incubation 1 hour at 37^0^C, 5%CO_2_. Cells were washed and kept in washing buffer (PBS supplemented with 2% FCS and 2mM EDTA). To determine activation status of pSTAT5 by flow cytometry, GM-CSF-driven BMDCs cells were cultured in the presence of 2% GM-CSF up to day 4 followed by overnight starvation with DMEM supplemented with 2% FCS. Subsequently, pre-sorted BMDCs (CD11c^+^CD11b^+^) were treated with 4% GM-CSF 5 minutes fixed with paraformaldehyde (PFA) and permeabilized with 90% cold methanol for 30 minutes. Subsequently cells were incubated AlexaFluor 488-conjugated pSTAT5 Ab one hour on ice, washed, and analyzed by flow cytometry.

### Real-Time PCR

Total RNA was extracted from GM-CSF-derived BMDCs or draining popliteal lymph nodes of immunized mice. For evaluation of mRNA transcripts of IL-12p40, IFNβ, IRF3, and IRF8, cDNA was generated using SuperScript III accordingly to the manufacturer’s instructions followed by Real-Time PCR. The following primer set was used: for IL-12p40: 5′ ACA GCA CCA GCT TCT TCA TCA G 3′ and 5′ TCT TCA AAG GCT TCA TCT GCA A 3’; for IRF8: 5′ CGG GGC TGA TCT GGG AAA AT 3′ and 5′ CAC AGC GTA ACC TCG TCT TC 3′; for GM-CSF 5′ GGC CTT GGA AGC ATG TAG AGG 3′ and 5′ GGA GAA CTC GTT AGA GAC GAC TT 3′; for IRF3: 5′ GAG AGC CGA ACG AGG TTC AG 3′ and 5′ CTT CCA GGT TGA CAC GTC CG 3′; for IFNβ: 5′ CAG CTC CAA GAA AGG ACG AAC 3′ and 5′ GGC AGT GTA ACT CTT CTG CAT 3′; for β-actin: 5′ GAA GAG CTA TGA GCT GCC TGA 3′ and 5′ GCA CTG TGT TGG CAT AGA GGT 3′

### Antigen presentation assay

CD11c^+^CD8α^+^ dendritic cells were sorted sequentially with antibody-coated magnetic beads and flow cytometry. Purifed cells were pulsed with different doses of SIINFEKL followed by co-incubation with OT-1 cells as described previously [7] to evaluate cell proliferation by thymidine incorporation assay. Alternatively, mice were immunized with SIINFEKL and CFA and popliteal lymph nodes were harvested 48 hrs later, treated with collagenase D for 1 hour followed by enrichment by positive selection (CD11c) with antibody-coated magnetic beads according to the manufacturer’s instruction. The CD11c^+^CD8α^+^ cell population was enriched further with flow cytometric sorting (FACSAria) and 2×10^3^ dendritic cells were co-incubated with 2×10^5^ OT-1 cells overnight to evaluate IFNγ production by ELISPOT.

### Clodronate liposomes depletion of monocytes and CCR2 inflammatory monocytes ablation

The depletion of phagocytic antigen presenting cells along with monocyte/macrophages with clodronate liposomes has been described [17]–[19]. Briefly, mice were injected i.p. every two days with 200 µl of clodronate liposomes (5 mg/ml) suspension, starting 2 days before immunization and continuing up to day 6 post immunization. For depletion of inflammatory monocytes expressing CCR2, the MC21 antibody was administered as previously described [19]. Briefly, mice were injected i.p. with MC21 Ab a day before immunization of SIINFEKL and CFA. Injection with the same dose of Ab was repeated daily up to day 4 post-immunization. Mice were sacrifice at day 7 and CD8 T cell response were measured in recall response assays using an ELISPOT for IFNγ.

### Statistical analysis

Results were analyzed with Graph Prism software (La Jolla, CA USA). Each experiment was repeated three times including 3–5 mice. All results were considered as significant if a P<0.05 was achieved using Student t-test**.**

## Results

### ITAM signaling negatively regulates CD8 T cell priming *in vivo*

**Figure 1:**
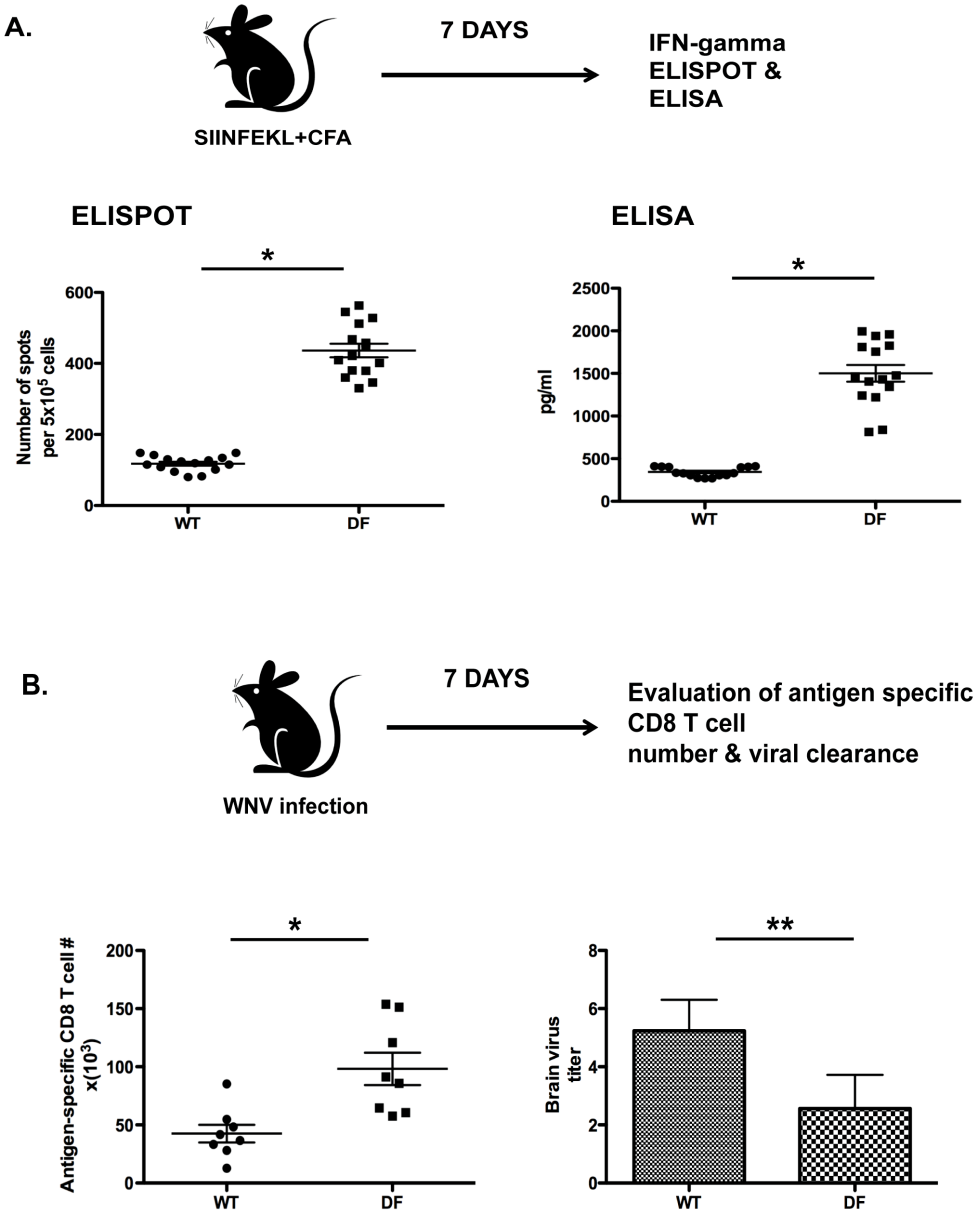
ITAM signaling negatively regulates the antigen response of CD8 T cells and clearance of WNV *in vivo.* (A) DF and WT mice were immunized with SIINFEKL and CFA. Seven days later endogenous CD8 T cell response from draining popliteal lymph nodes was measured either with ELISPOT or bead-based ELISA. Data are representative from three independent experiments including in total 30 mice (n = 15 in each group). (B) Mice were infected with WNV and antigen-specific CD8 T cell response was followed by virus clearance and analyzed seven days later. Data are representative from 3 independent experiments. *P<0.01, **P<0.05.

To determine the requirement for DC-ITAM signaling in CD8 T cell priming *in vivo*, we initially performed a series of immunization experiments. Dap12^−/−^ x FcRγ^−/−^ double KO mice (referred to DF mice) were immunized in the footpad with MHC class I restricted peptide (OVA_257-264_ SIINFEKL) and complete Freund’s Adjuvant, and a recall response of endogenous CD8 T cells in draining lymph nodes of WT and DF mice was measured seven days later using either ELISPOT or ELISA. These experiments revealed marked upregulation (4-folds, P < 0.01) of the endogenous CD8 T cell response in DF mice as compared to WT (Fig. 1A). To further explore this finding, we used a model of West Nile virus (WNV) infection in which CNS infection is mitigated by effector CD8 T cells [20]. We inoculated mice with WNV to determine the total number of antigen-specific CD8 T cells and virus titer in brain homogenates during primary infection. We found significantly higher numbers (2-fold, P <0.01) of antigen-specific CD8 T cells and decreased WNV titer (2-fold, P <0.05) in brain tissue in DF mice as compared to WT (Fig. 1B). Collectively, these results indicate enhanced CD8 T cell priming in DF mice, however the cell autonomy of these effects remained to be elucidated. Given that Dap12 and FcRγ are not required for T cell development and/or function [1], we focused our attention on dendritic cell subsets required for priming and differentiation of CD8 T cells.

### ITAM signaling is not required for regulation of CD8 T cell response by CD8α DCs *in vivo* and *in vitro*

**Figure 2:**
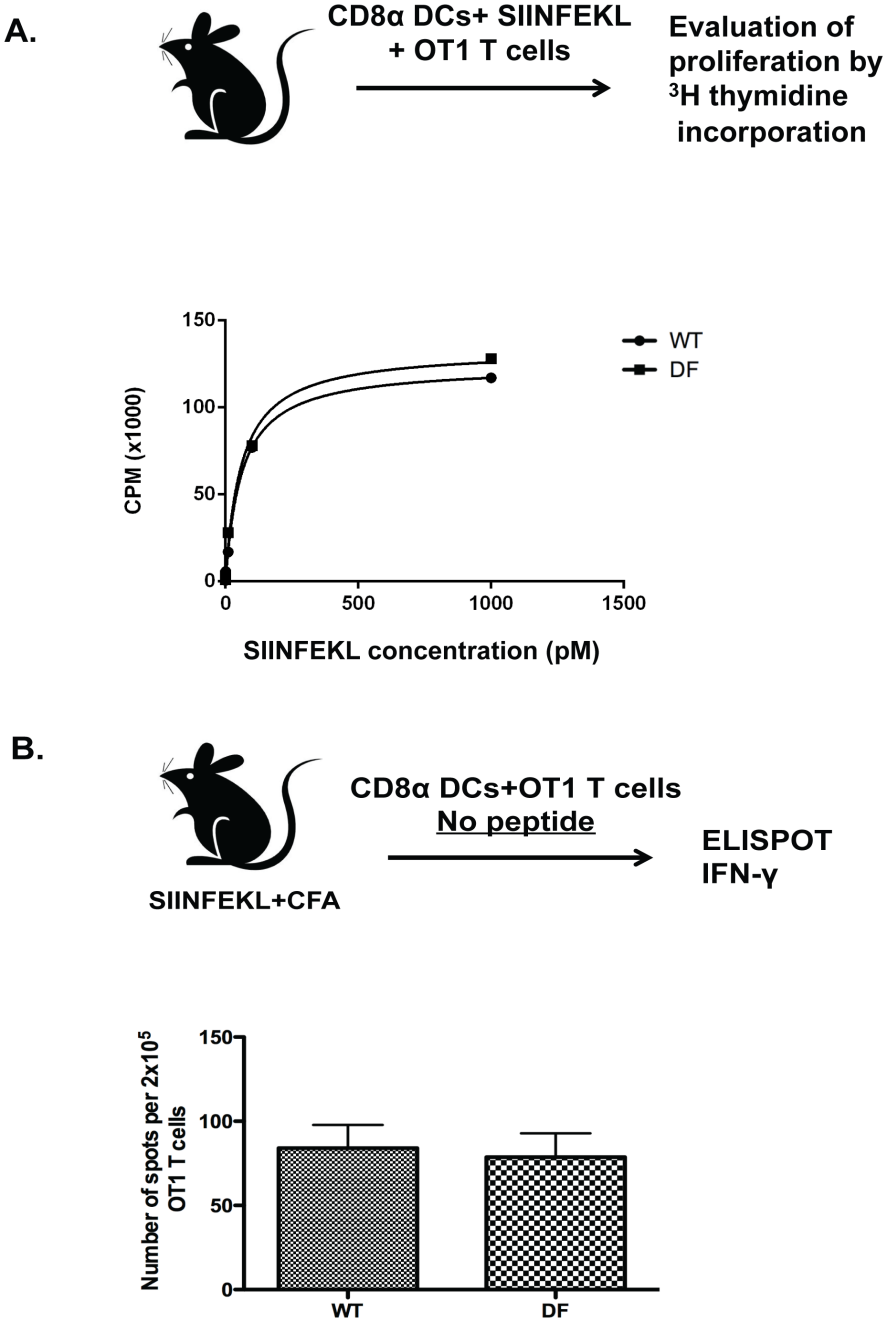
DC-ITAM signaling is not required for regulation of CD8 T cell priming by CD8α DCs. (A) CD8α DCs were isolated from naive DF and WT mice, pulsed with SIINFEKL peptide and co-cultured with OT1 T cell to evaluate T cell proliferation. (B) DF and WT mice were immunized with SIINFEKL and CFA into the footpad and CD8α DCs were isolated from draining popliteal lymph nodes by flow cytometry sorting. Isolated CD8α DCs were cultured *ex vivo* with OT1 T cells without exogenously added peptide to evaluate frequency of OT1 cells producing IFNγ with ELISPOT. Data are representative from three independent experiments including 3 mice per each investigated group.

To determine the cell autonomy of enhanced CD8 T cell response in DF mice, we isolated CD8α DCs from spleens of WT or DF mice. Next, we co-cultured them with OT1 ovalbumin-specific transgenic T cells. We analyzed proliferative response of OT1 cells and found no significant differences (P >0.05) between WT and DF (Fig. 2A). As a second approach, we immunized DF and WT mice with SIINFEKL and CFA using a standard immunization protocol and, after 48 hrs, isolated CD8α DCs from a draining lymph node of an immunized mouse by direct flow cytometric cell sorting. These cells were then cultured with OT1 T cells *in vitro* without any exogenously added peptide. We measured the frequency of OT1 T cells producing IFNγ and found no differences between DF and WT CD8α DCs (Fig. 2B). Collectively, these experiments showed that ITAM signaling is not required for regulating protein antigen presentation by CD8α DC.

### Loss of Dap12 and FcRγ leads to increased GM-CSF-driven differentiation of Mo-DCs

**Figure 3:**
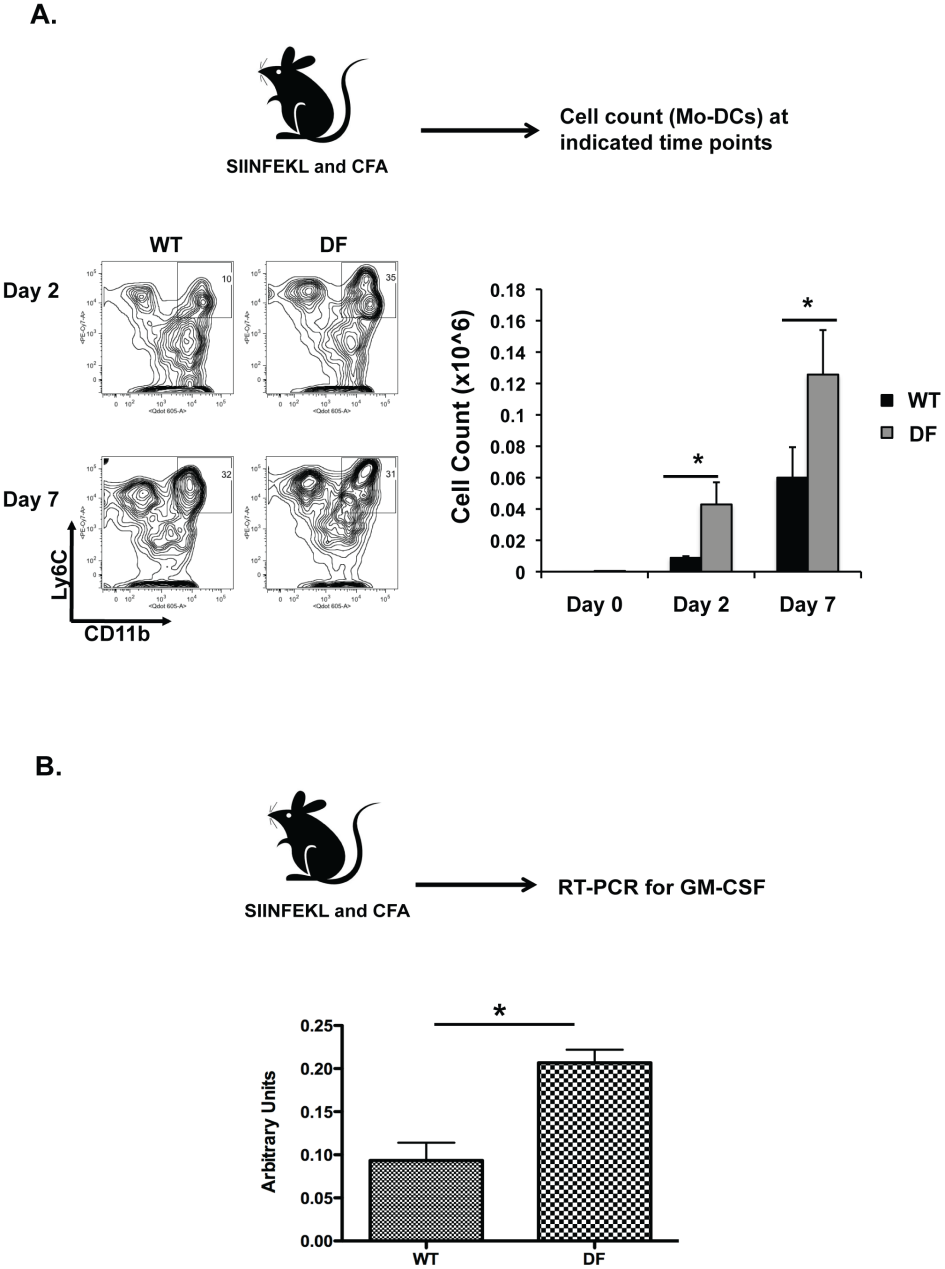
Increased expansion of Mo-DCs under inflammatory conditions is GM-CSF derived. (A) Mice were immunized in the footpad with SIINFEKL and CFA and frequencies and total number of Mo-DCs were evaluated at the indicated time points. (B) Mice were immunized in the footpad with SIINFEKL and CFA and the transcript levels of GM-CSF in the draining lymph nodes were evaluated at day 7 post-immunization. Data are representative from three independent experiments including 3 mice per each investigated group. *P<0.01

**Figure 4:**
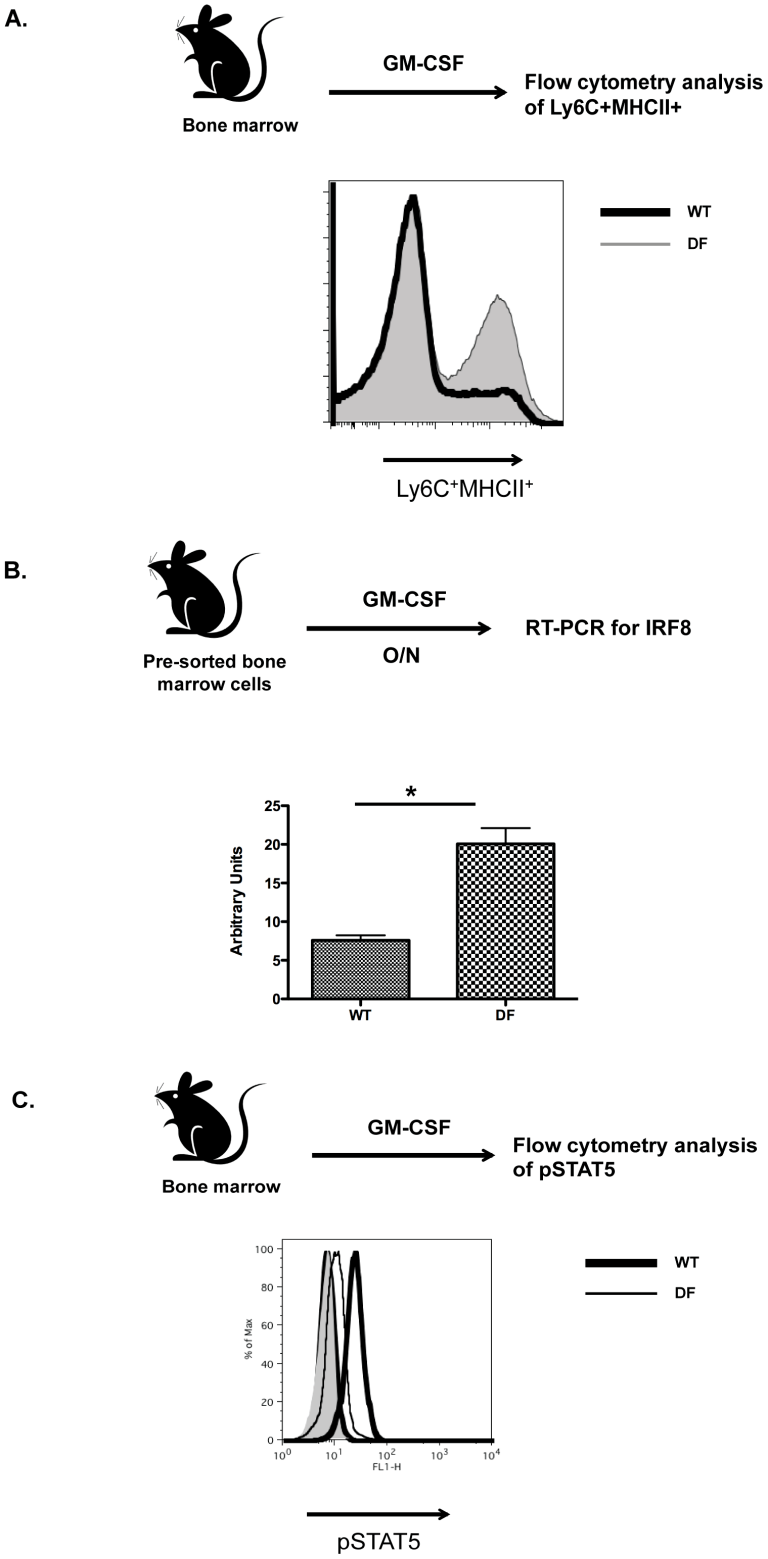
DC-ITAM signals negatively regulate GM-CSF-induced Mo-DCs differentiation. (A) Bone marrow from DF and WT mice were cultured in GM-CSF and BMDCs expansion (gated on CD11c^+^CD11b^+^) of Ly6C^+^MHCII^+^ was evaluated three days later. (B) Bone marrows cells from DF and WT were sorted by flow cytometry to obtain CD11b^+^Ly6C^+^ cells, which were then stimulated overnight with GM-CSF. Cells then were lysed and RT-PCR was used to evaluate IRF8 mRNA levels (normalized to β-actin). (C) BMDCs from DF and WT mice were cultured in the presence of GM-CSF up to 4 days. Cells then were placed in GM-CSF-deficient medium overnight, and then stimulated for 5 minutes with 4% GM-CSF followed by evaluation of intracellular pSTAT5 expression. All results are representative of 3 independent experiments encompassing 3 animals per each group. *P<0.01.

Given that the increased CD8 T cell priming in DF mice was not related to impaired function of CD8α DCs in the context of antigen presentation, nor was the number of CD8α DCs significantly altered (data not shown), we hypothesized that involvement of another DC subset might be responsible for de-regulation of CD8 T cell responses. In this context, we found that the immunization of DF and WT mice differed with respect to numbers of Mo-DC in draining lymph nodes, with the largest difference noted at day 7 post-immunization (Fig. 3A). In agreement with recently published data [21], we speculated that this effect might be GM-CSF-driven, as expression of this cytokine was significantly increased at day 7 post-immunization (Fig. 3B). To test this, we analyzed differentiation of WT and DF purified monocytes from bone marrow in the presence of GM-CSF and found markedly increased numbers of CD11c^+^CD11b^+^Ly6C^+^MHCII^+^ cells in DF as compared to WT mice (Fig. 4A). Previous studies showed that mice deficient for components of the ITAM signaling module (DF or Vav^NULL^) had altered responses to TLR triggering [4], [22]. In this context, freshly sorted CD11b^+^Ly6C^+^Ly6G^−^ bone marrow cells from DF and Vav^NULL^ mice stimulated overnight with GM-CSF showed increased expression of IRF8 mRNA in DF and Vav^NULL^ mice compared to WT mice (Fig. 4B and data not shown). Given that GM-CSF signals through STAT5 to suppress IRF8 expression [23], we tested the activation status of STAT5 in GM-CSF stimulated BMDCs from DF and Vav^NULL^ mice. Notably, we observed reductions in GM-CSF induced STAT5 phosphorylation (pSTAT5) in both DF and Vav^NULL^ as compared to WT mice (Fig. 4C and Fig. S1). Taken together, the increased number of Mo-DCs at the site of inflammation in DF mice might be caused at least in part by de-repression of IRF8 expression as a result of attenuated GM-CSF induced Stat5 phosphorylation.

### Loss of Dap12 and FcRγ leads to increased IL-12 production in GM-CSF-driven BMDCs

**Figure 5:**
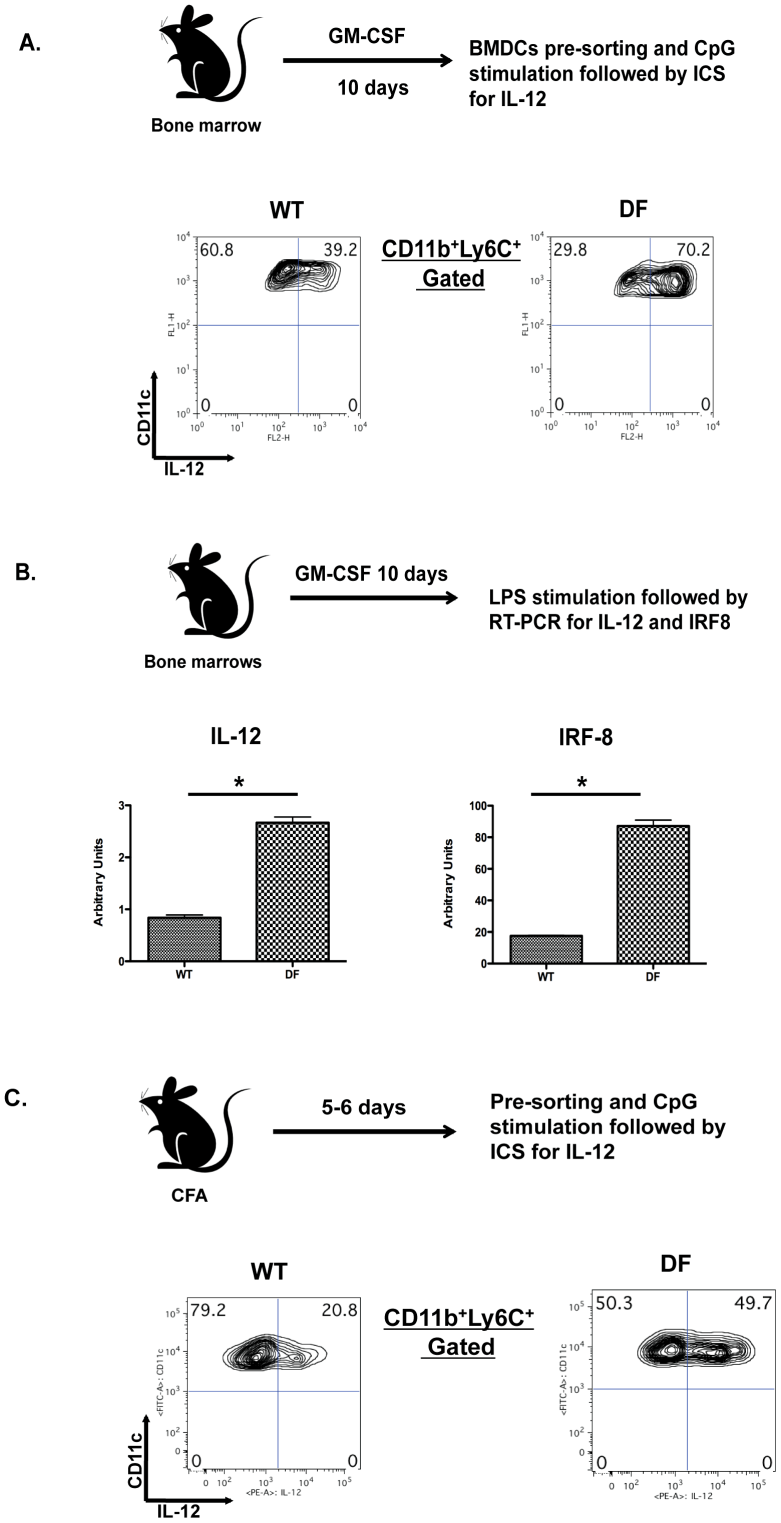
DC-ITAM deficiency show exaggerated production of IL-12 after TLR ligation. (A) Bone marrow from DF and WT mice were cultured in GM-CSF and at day 10 stimulated with CpG1826 (1 µM) for 6 hrs to evaluate levels of intracellular IL-12. (B) Bone marrow from DF and WT mice were cultured in GM-CSF and at day 10 pre-sorted CD11c^+^CD11b^+^ were stimulated with LPS (10ng/ml). Cells then were lysed and RT-PCR was used to evaluate IRF8 and IL-12 mRNA levels (normalized to β-actin). (C) Pre-sorted CD11c^+^ from draining lymph nodes of immunized DF and WT mice were stimulated *in vitro* with CpG1826 (1 µM) to evaluate intracellular IL-12 expression in CD11c^+^CD11b^+^Ly6C^+^MHCII^+^ cells. ICS – intracellular cytokine staining. Data are representative from three independent experiments including 3 mice per each investigated group.

Given that IRF8 promotes IL-12 expression [24] and Mo-DCs are a major source of this cytokine [4], [25], we first tested the ability of DF BMDCs to produce IL-12 in response to stimulation with CpG1826 (CpG-DNA). We observed a significant difference in production of IL-12 (P<0.01) between DF and WT mice in GM-CSF-derived BMDCs (Fig. 5A), results that are consistent with published literature [4]. Moreover, IL-12 expression correlated with IRF8 but not with IRF5 expression (Fig. 5B and data not shown). We also noted increased IL-12 secretion in BMDCs from DF compared to WT mice after either LPS or CpG-DNA stimulation (Fig. S2) and increased expression of IL-12 by DF Mo-DCs from lymph nodes of immunized mice. Thus, we immunized mice with CFA, and 5-6 days later, sorted CD11c^+^ cells from draining lymph nodes were stimulated *in vitro* with CpG-DNA to evaluate IL-12 synthesis within Mo-DCs. Consistent with our previous results, a dramatic increase was seen in IL-12 production by stimulated Mo-DCs from DF compared to WT mice (Fig. 5C). Importantly, ITAM signaling appears dispensable for regulation of IL-12 production in conventional DCs (Fig. S3 and Fig. S4). These findings suggest differential regulation of IL-12 production by ITAM signaling in Mo-DCs versus CD8α DCs, perhaps reflecting selective effects of GM-CSF on Mo-DCs. Thus, an ITAM deficiency results in increased IL-12 production specifically in Mo-DCs, suggesting that this DC population might regulate endogenous CD8 T cell priming after immunization.

### Mo-DCs regulate endogenous CD8 T cell responses

**Figure 6:**
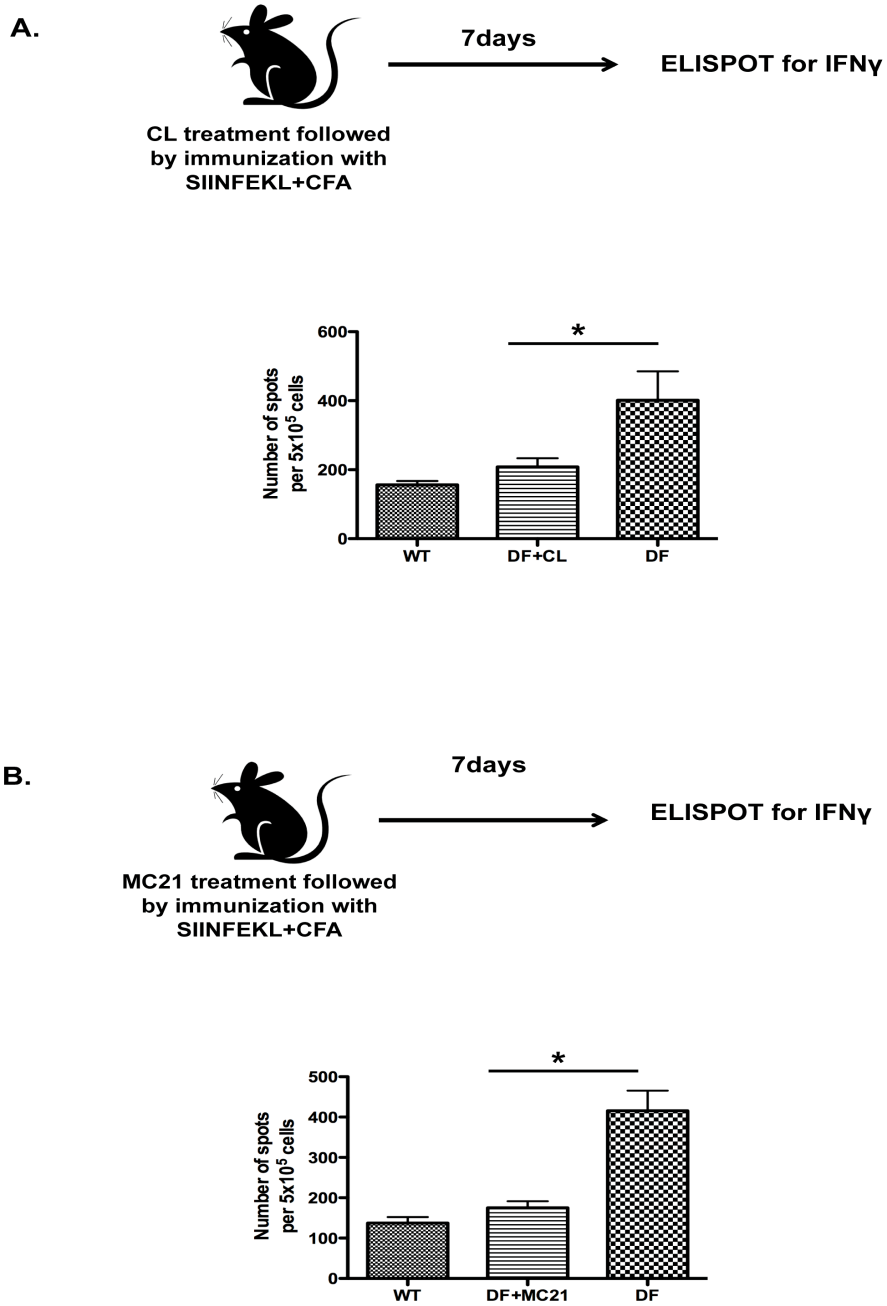
CCR2 inflammatory monocytes/Mo-DCs drive an exaggerated CD8 T cell responses in DC-ITAM deficient mice. (A) DF mice were injected i.p. every two days with 200 µl of clodronate liposomes (5 mg/ml) following footpad immunization with SIINFEKL and CFA and the endogenous CD8 T cell response were measured at day7 by ELISPOT. (B) DF mice were injected i.p. with MC21 Ab consecutively five days up to day 4 post-immunization. The endogenous CD8 T cell response was measured in a recall response at day 7 by ELISPOT. Data are representative from 3 independent experiments encompassing 3-5 mice per each group. *P<0.01.

To test the requirement of inflammatory monocyte/Mo-DCs in regulation of CD8 T cell priming, we used two approaches. In the first, we depleted all phagocytes (monocytes, macrophages and dendritic cells) from the bloodstream with >80% efficiency using clodronate liposomes (Fig. S5). These experiments revealed down-regulation of antigen-specific CD8 responses in DF mice as measured by frequency of CD8 T cells producing IFNγ (Fig. 6A). In a second approach, we depleted monocytes and Mo-DCs in a more specific manner. Given that inflammatory monocytes express CCR2 and are precursors of Mo-DCs, we treated DF mice i.p. with an anti-CCR2 depleting antibody (MC21) on consecutive days up to day 4 post-immunization with SIINFEKL peptide and CFA. The efficiency of CCR2^+^ monocyte depletion was confirmed by flow cytometry showing >80% reduction in frequency of CD115^+^Ly6C^+^ population in the bloodstream (Fig. S5). At day 7 after immunization, DF mice treated with anti-CCR2 showed a significant reduction in IFNγ producing endogenous CD8 T cells as compared to non-treated DF mice (Fig. 6B). Taken together, our data shows that ITAM signaling negatively regulates CD8 T cell responses by a mechanism dependent on Mo-DCs. Moreover, ITAM signaling limits Mo-DC differentiation in response to GM-CSF and IL-12 production in response to TLR ligation.

## Discussion

The function of ITAM signaling in myeloid cells remains incompletely understood, as ITAM pathways have been reported to either positively or negatively regulate immune responses, depending on the experimental setting. To assess the mechanisms of ITAM signaling *in vivo*, we utilized mice lacking two ITAM-containing adapters Dap12 and FcRγ. Given that T cell development and function appear unaltered in these mice [8], they provide a suitable model to study the role of ITAM signaling in dendritic cells. We observed increased CD8 T cell priming in Dap12 and FcRγ deficient mice, which is not due to altered antigen presentation or loading by CD8α DCs. Instead we demonstrate that upregulation of CD8 T cell responses is related to altered function of Mo-DCs, which are major cellular sources of IL-12. Thus, the Mo-DCs dependent inflammatory *milieu* regulates CD8 T cell activation and differentiation in mice deficient for ITAM-containing adaptors.

Our study indentified Mo-DCs as a population that is recruited to inflamed lymph nodes and shapes the magnitude of endogenous CD8 T cell responses. This process is CCR2-dependent but does not require CCL2 and CCR7 [26]. Consistent with this, the mice deficient in CCR2 showed defective T cell responses and impaired production of Th1- type cytokines [27]. By using an antibody specifically targeting CCR2 on monocytes/Mo-DCs, we recapitulated the CCR2^−/−^ phenotype: DF mice treated with this antibody showed reduced CD8 T cell responses, that were comparable to that in WT mice. Thus, our results further support the role of Mo-DCs in regulating adaptive immune responses [26] and are consistent with studies showing that CCR2^+^ monocytes/Mo-DCs limit viral, bacterial or fungal diseases [28]. Given the significance of cross-talk between ITAM and TLR signaling [4], [9] as well as the importance of TLRs in restricting WNV infection [29], ITAM signaling in myeloid cells may be required for protection against WNV by regulating of cell migration and/or cytokine production. Importantly, myeloid cells from TLR7^−/−^ [30] but not TLR3^−/−^ [31] mice infected with WNV showed defects in inflammatory cytokine production, suggesting a role for MyD88 [29], which is also required for IL-12 production in Mo-DCs [25]. IL-12 is known to protect against WNV as mice deficient in IL-12p40 show are vulnerable to lethal infection [30]. While our studies do not address the specific role of Mo-DCs during WNV infection in DF mice, previous studies have shown a protective role for CCR2 in CNS infiltration by Ly6C^high^ monocytes in WT mice [32]. Accordingly, we speculate that ITAM signaling in inflammatory monocytes limits monocytosis and cytokine-mediated enhancement of CD8 T cell responses against WNV.

The exact mechanism concerning the massive accumulation of DF Mo-DCs after footpad immunization remains to be elucidated, as it might be caused either by regulation of migration [33], retention [34], or differentiation process [35]. Our results provide evidence that GM-CSF-driven differentiation of Mo-DC precursors is regulated by DC-ITAM signaling. The GM-CSF response was up-regulated in DF mice, which enhanced differentiation of Mo-DCs under inflammatory conditions. Indeed, treatment of mice with GM-CSF or transgenic expression of GM-CSF also enhanced expansion of Mo-DCs [21]. Our study suggests that ITAM pathways engage in a cross-talk with the GM-CSF pathway. Indeed, FcRγ physically associates with one of the GM-CSF receptor subunits; the common β chain (βc) and regulates cytokine production in basophils [36]. In this context, our data show that a complete ITAM deficiency in BMDCs from DF mice leads to a defect in GM-CSF induced STAT5 phosphorylation and IRF8 expression. As a combined Dap12- and FcRγ-deficiency augments GM-CSF-driven differentiation and production of inflammatory Mo-DCs, the STAT5-IRF8 axis appears critical for regulation of Mo-DC differentiation.

A positive feedback loop exists between IL-12 and IFNγ in which IL-12 augments IFNγ production in T cells, and conversely, IFNγ enhances IL-12 production in DCs. CD8α DCs are believed to be the initial producers of IL-12 during inflammatory conditions, and in this context, production of IL-12 is IFNγ independent [37]. However, recent data showed that while CD8α DC subset is a major producer of IL-12 in steady state conditions, Mo-DCs may be the critical producer of IL-12 during inflammatory conditions [25]. Importantly, in our experimental system, DC-ITAM signaling was dispensable for regulating IL-12 production by conventional DCs but not in Mo-DC. The reason for distinct regulation of IL-12 production between CD8α DCs and Mo-DCs in DF mice during inflammatory conditions requires further study. It is possible that cross-talk between ITAM and GM-CSF-signaling that drives Mo-DC differentiation may “pre-establish” an expression pattern of transcription factors that enhances the inflammatory profile after pathogen exposure [38]. Importantly, GM-CSF-driven IRF8 expression is further stimulated by IFNγ and TLR stimulation [24], [39]. As IRF8 is critical for regulating the cross-talk between TLR and IFNγ signaling [24], it also may modulate cell trafficking through regulation of CCR2 expression [40]. Moreover, IRF8 was identified as a downstream target for Notch-RBP-J pathway and TLR signaling, thus further supporting its role in macrophage proinflammatory polarization [41]. Given that IRF8-IL-12 expression correlates with expression of IRF3 and IFNβ (Fig. S6) it is plausible that IRF8 contributes to enhanced type I IFN response [42] in Mo-DCs in DF mice via a positive feedback loop. Overall, our results show that GM-CSF-induced IRF8 expression during Mo-DC differentiation is required for regulation of CD8 T priming. In the absence of DC-ITAM signaling, Mo-DCs exhibit increased capacity to produce IL-12 leading that results in an exaggerated CD8 T cell effector response.

## Supporting Information

Figure S1 **pSTAT5 expression is abrogated in Vav^NULL^ GM-CSF-induced BMDCs.** Bone marrow cells from Vav^NULL^ and WT mice were expanded in GM-CSF up to day 4 followed by overnight GM-CSF starvation. BMDCs then were stimulated at the indicated time points with 4% GM-CSF, and cell lysate were separated by 10% SDS-PAGE. Western blotting was performed with a phospho-specific antibody against STAT5. Membranes that were probed with pSTAT5 Ab were stripped with Western Blot stripping buffer (made in-house) and re-probed with Erk2. Data are representative from 3 independent experiments including 2–3 animals per each group.
(TIF)

Figure S2 **IL-12 production after LPS or CpG stimulation.** Bone marrow cells from DF and WT mice were expanded in GM-CSF up to day 10. After sorting of CD11c^+^CD11b^+^ BMDCs, cells were stimulated either with CpG1826 (250 nM) or LPS (10 ng/ml) for 6hrs and supernatants were evaluated for IL-12p40 levels using IL-12p40 FlexSet beads (bead-based ELISA). Data are representative from 3 independent experiments encompassing 3–4 animals per each group. *P<0.01.
(TIF)

Figure S3 **IL-12 production in Flt3L-driven BMDCs in DF and WT mice.** Bone marrow cells from DF and WT mice were expanded in Flt3L up through day10. Subsequently, BMDCs were stimulated with CpG1826 (1 µM) 6hrs. Subsequently, the CD11c^+^PDCA1^−^ cells were monitored for intracellular IL-12 cytokine levels. Data are representative from 3 independent experiments including 3 mice per each group.
(TIF)

Figure S4 **IL-12 production in conventional DCs stimulated ex vivo.** DF and WT mice were immunized with CFA in the footpad and after 5 to 6 days the popliteal lymph nodes were harvested, treated with collagenase D and sorted for CD11c^+^ DCs. Sorted cDCs (CD11c^+^CD8α^+^) were stimulated with CpG1826 (1 µM) 6hrs and stained for IL-12. Representative data from 3 independent experiments including 3 mice per group was shown.
(TIF)

Figure S5 **Efficiency of myeloid cell and CCR2+ monocytes depletion after treatment with clodronate liposomes (CL) or MC21 Ab.** DF mice were treated with either clodronate liposomes (CL) or MC-21 Ab followed by footpad immunization with SIINFEKL and CFA. Cell depletion was evaluated at day 6 (CL) or day 5 (MC-21). Data are representative from 3 independent experiments including 3 mice per each group.
(TIF)

Figure S6 **Loss of Dap12 and FcRγ negatively regulates IRF3-IFNβ axis in GM-CSF-derived BMDCs.** Bone marrow cells from DF and WT mice were expanded in GM-CSF up through day 10 followed by pre-sorting of CD11c^+^CD11b^+^ BMDCs. Subsequently, BMDCs were stimulated with LPS (10 ng/ml) overnight. Cells were lysed with using Trizol. cDNA was isolated with SuperScript III and RT-PCR was performed using SyberGreen along with specific primer set for either IRF3 or IFNβ. The representative result from 3 independent experiments encompassing 3 mice per group was shown. *P<0.01.
(TIF)
