# Supplementary figures and images for: Loss of DAP12 and FcRγ Drives Exaggerated IL-12 Production and CD8+ T Cell Response by CCR2+ Mo-DCs
Source: PLoS One. 2013 Oct 14;8(10):e76145. doi: 10.1371/journal.pone.0076145 (PMC3796521; doi:10.1371/journal.pone.0076145)

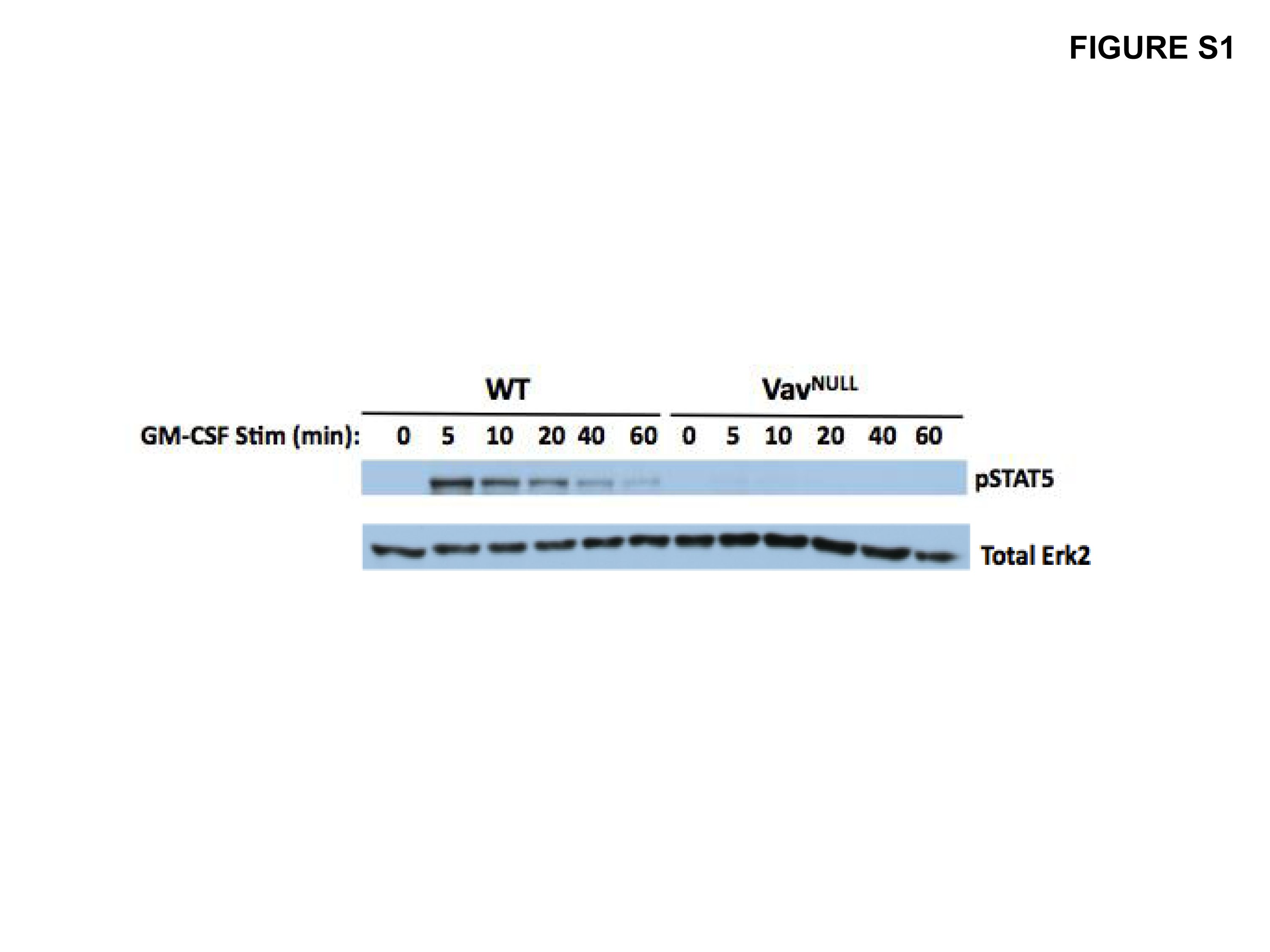

Supplement: Figure S1 — pSTAT5 expression is abrogated in VavNULL GM-CSF-induced BMDCs. Bone marrow cells from VavNULL and WT mice were expanded in GM-CSF up to day 4 followed by overnight GM-CSF starvation. BMDCs then were stimulated at the indicated time points with 4% GM-CSF, and cell lysate were separated by 10% SDS-PAGE. Western blotting was performed with a phospho-specific antibody against STAT5. Membranes that were probed with pSTAT5 Ab were stripped with Western Blot stripping buffer (made in-house) and re-probed with Erk2. Data are representative from 3 independent experiments including 2–3 animals per each group. (TIF) [file pone.0076145.s001.tif]

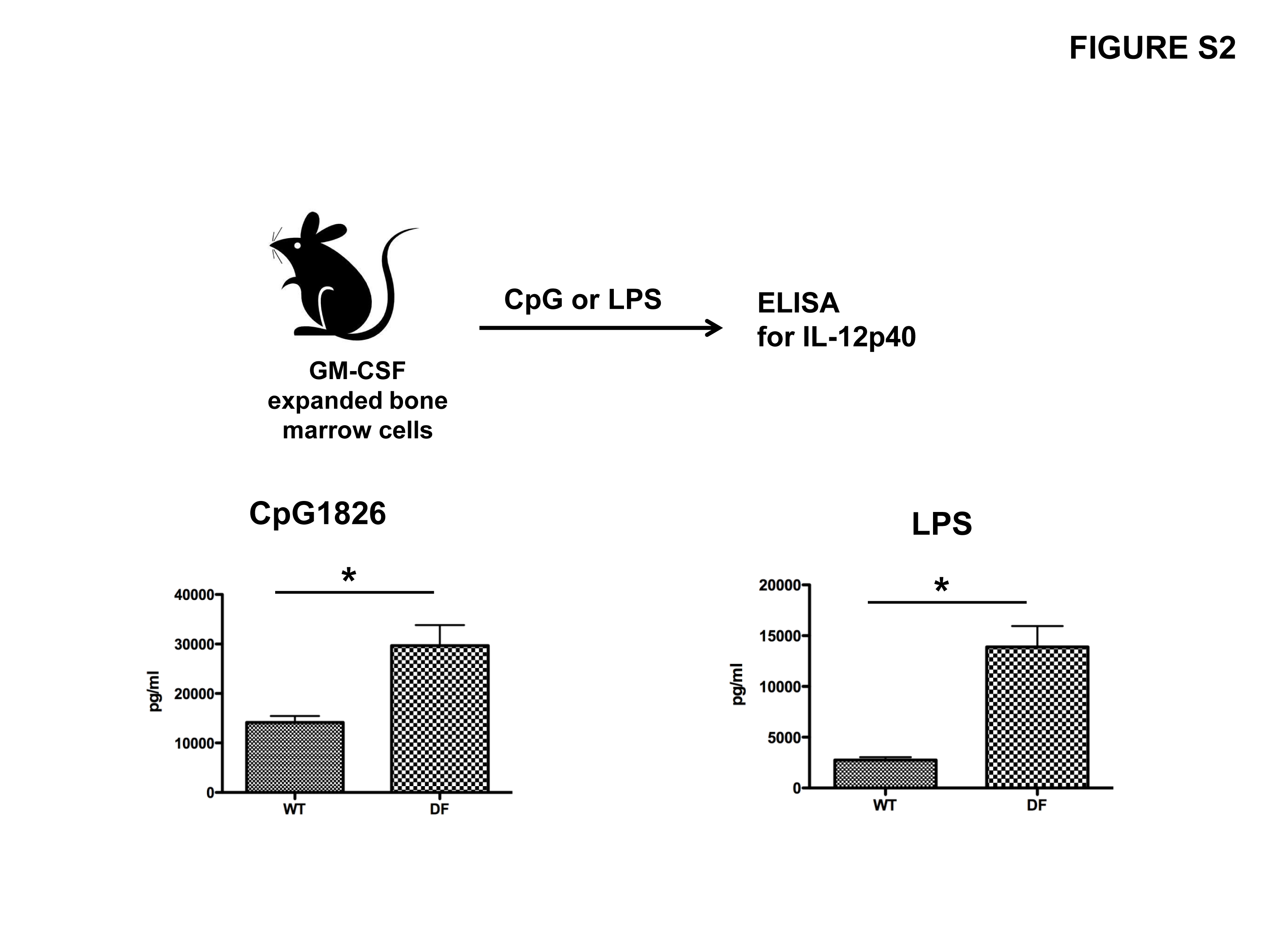

Supplement: Figure S2 — IL-12 production after LPS or CpG stimulation. Bone marrow cells from DF and WT mice were expanded in GM-CSF up to day 10. After sorting of CD11c+CD11b+ BMDCs, cells were stimulated either with CpG1826 (250 nM) or LPS (10 ng/ml) for 6hrs and supernatants were evaluated for IL-12p40 levels using IL-12p40 FlexSet beads (bead-based ELISA). Data are representative from 3 independent experiments encompassing 3–4 animals per each group. *P<0.01. (TIF) [file pone.0076145.s002.tif]

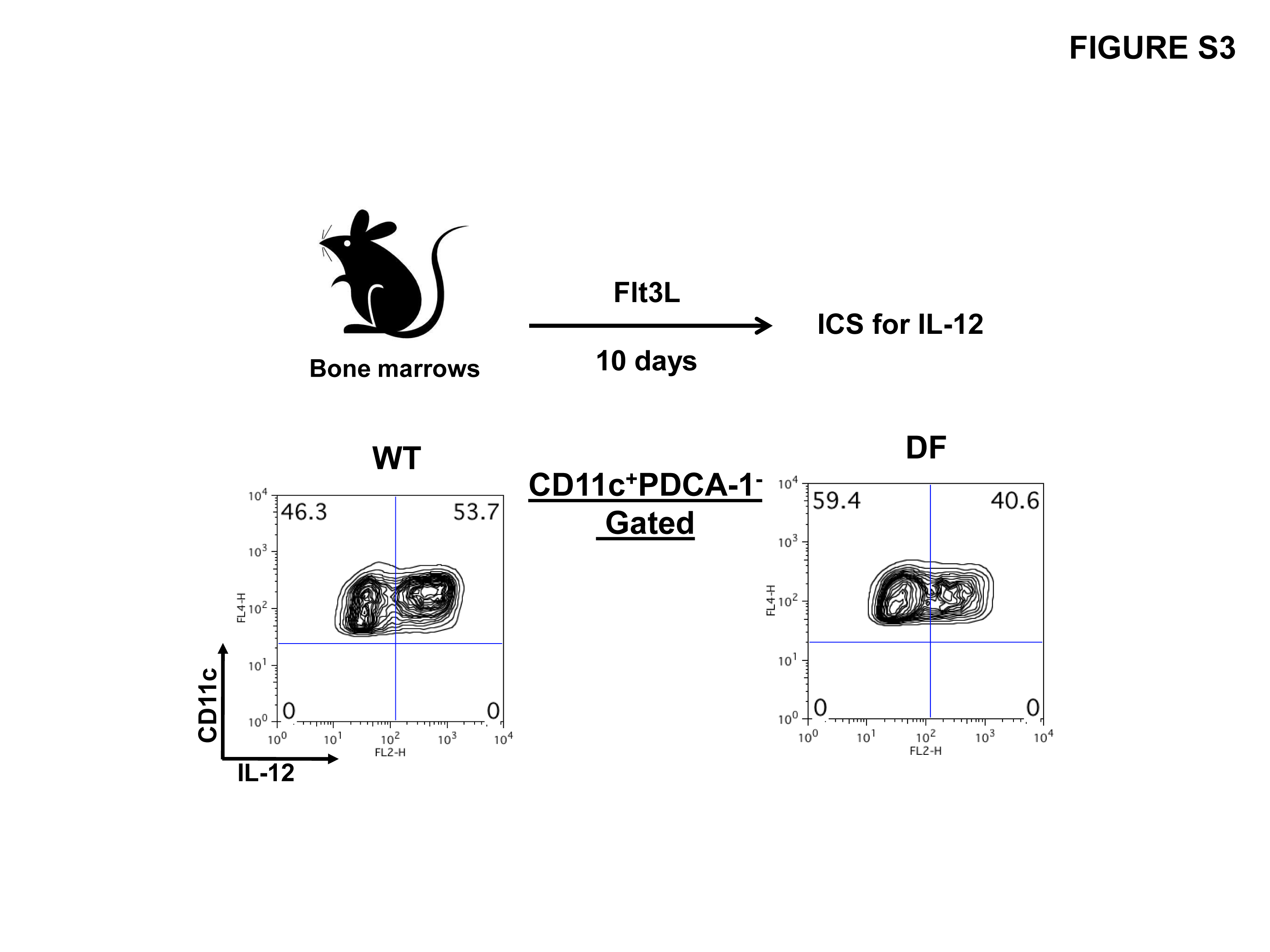

Supplement: Figure S3 — IL-12 production in Flt3L-driven BMDCs in DF and WT mice. Bone marrow cells from DF and WT mice were expanded in Flt3L up through day10. Subsequently, BMDCs were stimulated with CpG1826 (1 µM) 6hrs. Subsequently, the CD11c+PDCA1− cells were monitored for intracellular IL-12 cytokine levels. Data are representative from 3 independent experiments including 3 mice per each group. (TIF) [file pone.0076145.s003.tif]

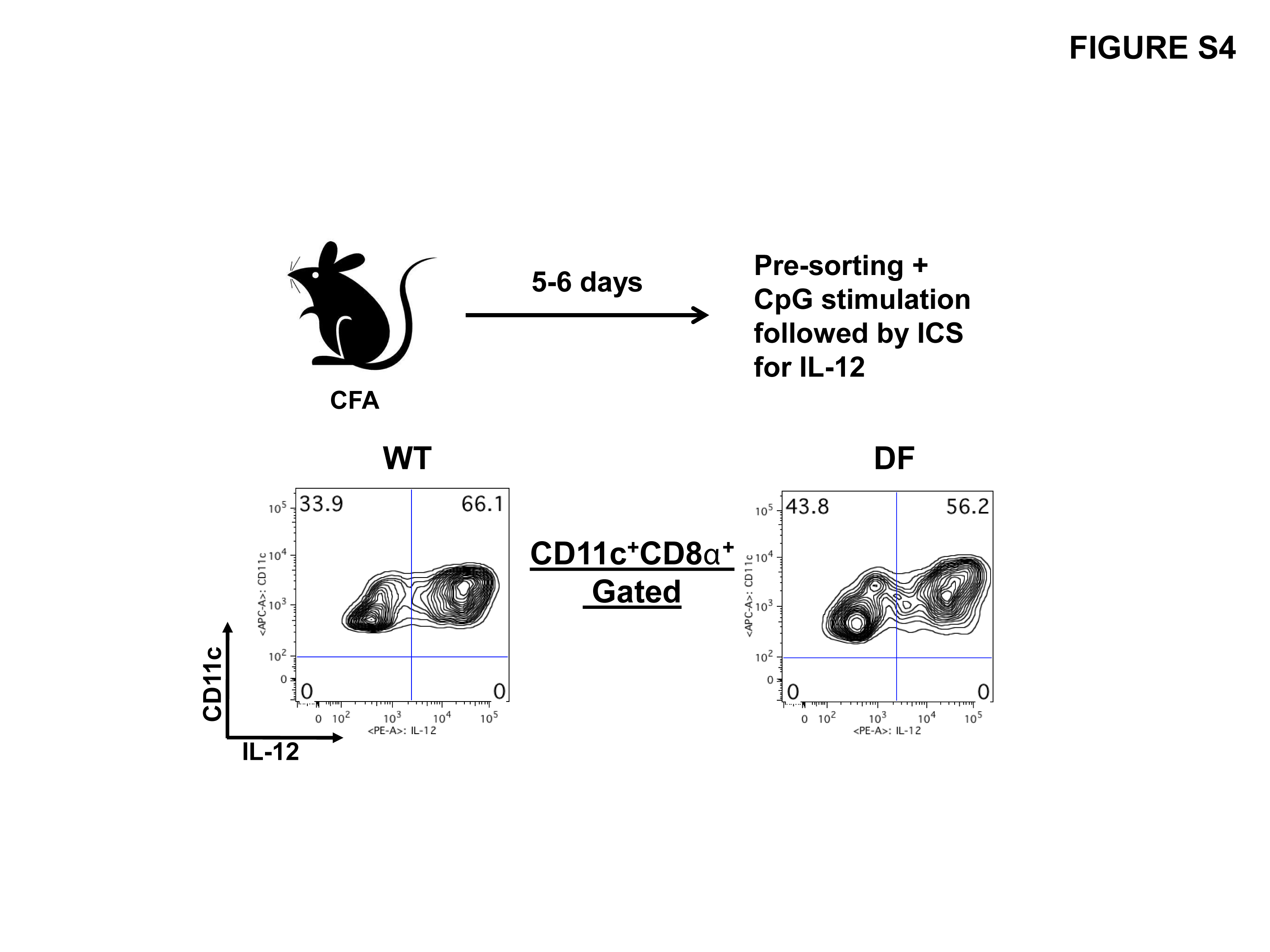

Supplement: Figure S4 — IL-12 production in conventional DCs stimulated ex vivo. DF and WT mice were immunized with CFA in the footpad and after 5 to 6 days the popliteal lymph nodes were harvested, treated with collagenase D and sorted for CD11c+ DCs. Sorted cDCs (CD11c+CD8α+) were stimulated with CpG1826 (1 µM) 6hrs and stained for IL-12. Representative data from 3 independent experiments including 3 mice per group was shown. (TIF) [file pone.0076145.s004.tif]

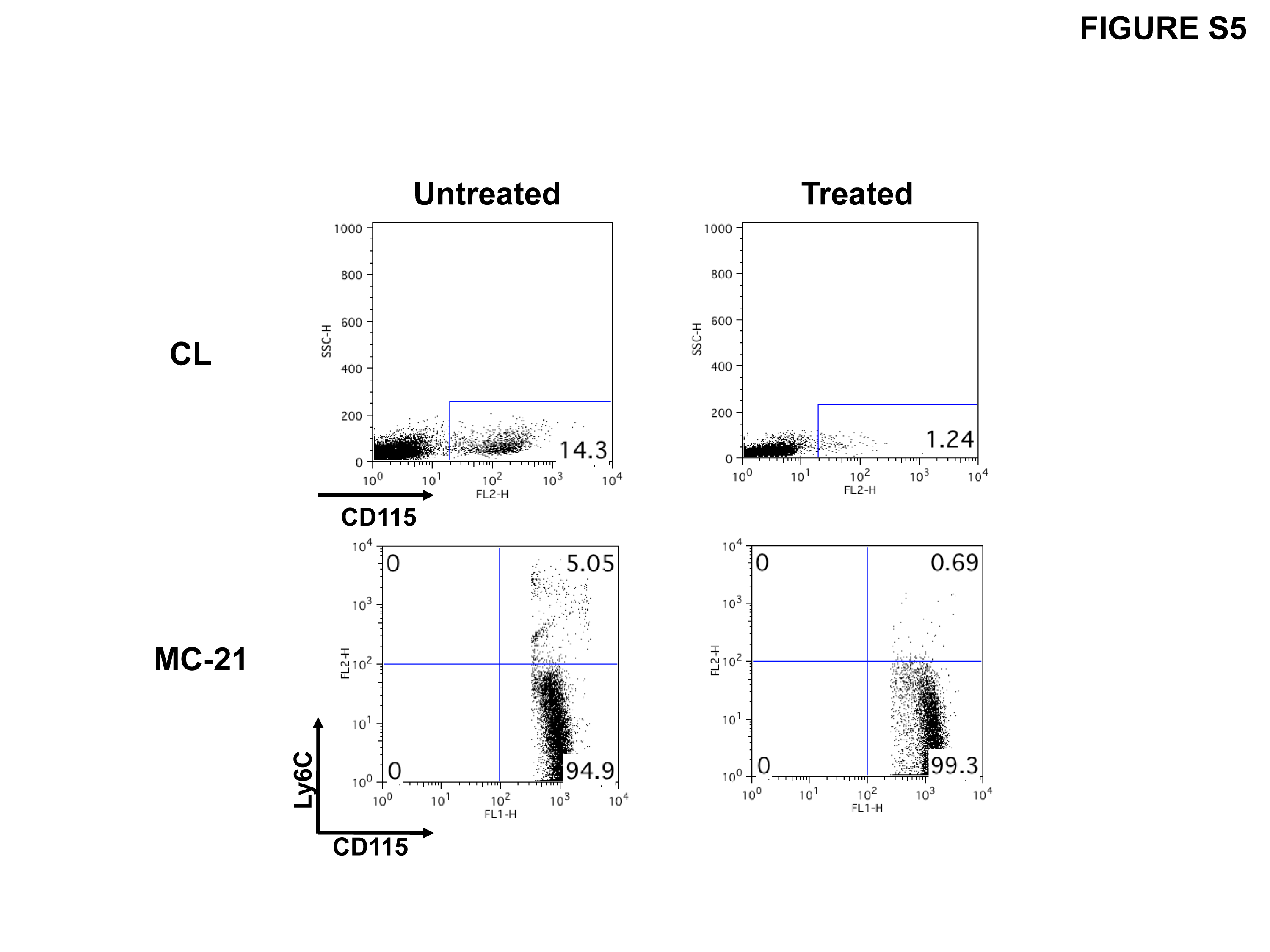

Supplement: Figure S5 — Efficiency of myeloid cell and CCR2+ monocytes depletion after treatment with clodronate liposomes (CL) or MC21 Ab. DF mice were treated with either clodronate liposomes (CL) or MC-21 Ab followed by footpad immunization with SIINFEKL and CFA. Cell depletion was evaluated at day 6 (CL) or day 5 (MC-21). Data are representative from 3 independent experiments including 3 mice per each group. (TIF) [file pone.0076145.s005.tif]

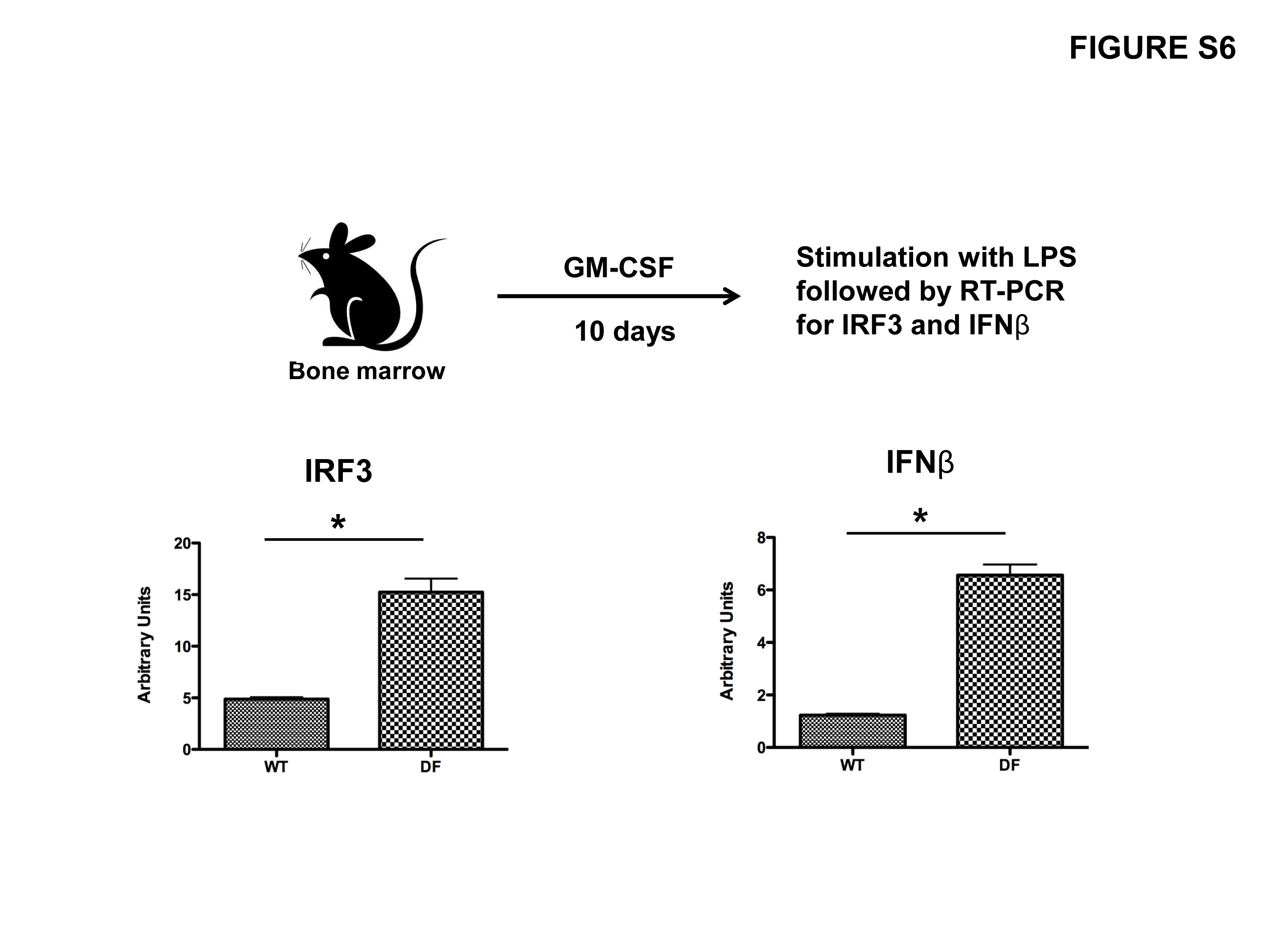

Supplement: Figure S6 — Loss of Dap12 and FcRγ negatively regulates IRF3-IFNβ axis in GM-CSF-derived BMDCs. Bone marrow cells from DF and WT mice were expanded in GM-CSF up through day 10 followed by pre-sorting of CD11c+CD11b+ BMDCs. Subsequently, BMDCs were stimulated with LPS (10 ng/ml) overnight. Cells were lysed with using Trizol. cDNA was isolated with SuperScript III and RT-PCR was performed using SyberGreen along with specific primer set for either IRF3 or IFNβ. The representative result from 3 independent experiments encompassing 3 mice per group was shown. *P<0.01. (TIF) [file pone.0076145.s006.tif]
